# Consumption of Ultra-Processed Foods and Semen Quality in Healthy Young Men Living in Italy

**DOI:** 10.3390/nu16234129

**Published:** 2024-11-29

**Authors:** Elisabetta Ceretti, Marialaura Bonaccio, Licia Iacoviello, Augusto Di Castelnuovo, Emilia Ruggiero, Francesco Donato, Stefano Lorenzetti, Danilo Zani, Luigi Montano

**Affiliations:** 1 Unit of Hygiene, Epidemiology and Public Health, Department of Medical and Surgical Specialties, Radiological Sciences and Public Health, University of Brescia, 25123 Brescia, Italy; francesco.donato@unibs.it; 2 Department of Epidemiology and Prevention, IRCCS Istituto Neurologico Mediterraneo NEUROMED, 86077 Pozzilli, Italy; marialaura.bonaccio@moli-sani.org (M.B.); licia.iacoviello@moli-sani.org (L.I.); dicastel@moli-sani.org (A.D.C.); emilia.ruggiero@moli-sani.org (E.R.); 3 Department of Medicine and Surgery, LUM University, 70010 Casamassima, Italy; 4 Department of Food Safety, Nutrition and Veterinary Public Health, Italian National Institute of Health (ISS), 00161 Rome, Italy; stefano.lorenzetti@iss.it; 5 Unit of Urology, Department of Medical and Surgical Specialties, Radiological Sciences and Public Health, University of Brescia, 25123 Brescia, Italy; danilo.zani@unibs.it; 6 Andrology Unit and Service of Lifestyle Medicine in UroAndrology, Local Health Authority (ASL) Salerno, “Oliveto Citra Hospital”, 84124 Salerno, Italy; luigimontano@gmail.com; 7 Coordination Unit of the Network for Environmental and Reproductive Health (EcoFoodFertility Project), “Oliveto Citra Hospital”, 84124 Salerno, Italy

**Keywords:** ultra-processed food, semen quality, reproductive factors

## Abstract

Background/Objectives: The study aim was to evaluate the association between UPF consumption and semen quality in a sample of healthy young men in Italy. Methods: A cross-sectional analysis was carried out using data from 126 participants (mean age ± SD 20.0 ± 1.2 years) enrolled in the FASt randomized controlled trial. Food intake was assessed through the European Prospective Investigation into Cancer and Nutrition (EPIC) FFQ. Food items were categorized according to the Nova classification based on their purpose and extent of processing as follows: (1) unprocessed/minimally processed foods; (2) processed culinary ingredients; (3) processed foods; and (4) UPFs. The weight ratio (%) between each Nova group (g/d) and total food (g/d) was then calculated. For semen analyses, sperm volume, concentration, motility and morphology were measured. The associations between UPF consumption (quarters of) and semen quality parameters were estimated using multivariable-adjusted linear regression models. Results: Participants consuming high UPFs (Q4), compared to those in the bottom category of intake (Q1), had a lower sperm concentration (β = −54.16 × 10^6^ cell/mL; 95%CI: −92.91 to −15.40; *p* for trend = 0.0020 across fourths) and progressive motility (β = −14.17%; 95%CI: −28.25 to −0.09; *p* for trend = 0.036). The percentage of normal morphology cells had a tendency to decrease amongst subjects consuming more UPFs compared to those with the lowest intake. Conclusions: A large dietary intake of UPFs was inversely associated with sperm concentration and progressive motility in reproductive-age men. These findings suggest that dietary recommendations for improving male fertility and sperm health should also recommend limiting UPFs.

## 1. Introduction

Male infertility is a significant public health issue, which affects approximately 9–15% of men worldwide, accounting for 30% of all infertility cases [1]. The prevalence of male infertility has increased since 1990 [2], with an estimated increasing rate of about 0.29% per year [3]. One of the most commonly used approaches to assess male infertility is the evaluation of semen quality, which includes various semen parameters [4]. In recent years, studies on male semen in several countries have shown a gradual decrease in male semen quality, mainly in sperm count [5,6,7].

Many physiological, behavioral and environmental factors have been associated with male infertility and semen quality, with different grades of evidence [8,9,10,11]. Among lifestyle factors, dietary habits seem to have a relevant impact on semen quality [12,13,14,15,16,17]. Several studies have investigated the role of individual nutrients and bioactive compounds, such as antioxidants or saturated and trans fats, in determining sperm quality. More recently, however, interest has been focused on dietary patterns, which seem to have a stronger impact on sperm quality than the consumption of single nutrients [18,19,20,21]. One of the most recently investigated pattern-related dietary factors is the consumption of ultra-processed foods (UPFs). According to the Nova classification, foods, food products and beverages can be classified into four groups based on the extent and purpose of the industrial processing they undergo [22]. UPFs are industrial formulations with a large number of ingredients (typically five or more) made of processed substances derived from whole foods, which are chemically modified and assembled, frequently using food additives (additives with cosmetic functions, such as colors, flavors, emulsifiers and sweeteners), in order to create “highly profitable (low-cost ingredients, long shelf-life, emphatic branding), convenient (ready-to-consume), hyper-palatable products” [23]. In recent decades, public concern has been raised about the health impact of UPFs, given the increasing volume of industrially processed food worldwide, with the gradual replacement of traditional diets. The consumption of processed and ultra-processed foods is higher in non-Mediterranean than in Mediterranean countries: it represents almost 60% of total energy intake in the USA and UK [24,25], 46% in Canada [26], 42% in Australia [27], 24% in Spain [28] and 17.3% in Italy [29].

UPF consumption has been associated with several different health outcomes in many studies conducted worldwide. High consumption of UPFs was linked to an increased risk of all-cause, cardiovascular and cancer mortality [30,31,32,33,34]. Accordingly, it was associated with an increasing risk of conditions and diseases, including being overweight or obese, metabolic syndrome, hypertension, inflammation, diabetes, asthma, cancer, cardiovascular diseases, depression and others [30,35,36,37,38,39,40].

Given the wide range of biological effects and diseases associated with UPF consumption, a possible role of these foods in reduced fertility has been hypothesized. Few studies have investigated the association between UPF consumption and semen quality so far, finding a lower sperm concentration and motility and higher odds of asthenozoospermia in males with a higher UPF intake [41,42,43]. However, more research is needed to draw a definite conclusion on this topic.

This study, part of a large, randomized trial [44], aimed to assess the association between UPF consumption and semen quality in a sample of healthy, normal-weight young men in Italy.

## 2. Materials and Methods

### 2.1. Study Population

A cross-sectional analysis was conducted using data from the FASt randomized controlled trial in 2018–2019. The FASt (Fertilità, Ambiente, alimentazione, STile di vita) study was a randomized controlled trial (clinicaltrials.gov Protocol Registration and Results System; receipt release date: 15 February 2019; No. J59D160013 2001) aiming to assess the effects of an intervention addressing diet and physical activity on the semen quality of healthy young men living in different areas of Italy, as detailed elsewhere [44,45]. Briefly, 18–22-year-old students from high school and university courses were selected in accordance with the following inclusion criteria: residence in one of the study areas; no varicocele surgery in the last 6 months; no history of chronic diseases; no regular use of drugs or medicine, alcohol or tobacco; no dietary supplement intake; and not overweight/obese (body mass index > 25 or waist circumference > 102 cm). Participants underwent urological examination, measurements of weight, height and abdominal circumference and an interview on lifestyle variables. Questionnaires for evaluating the frequency of food intake (EPIC questionnaire) [46,47] and physical activity (IPAQ questionnaire) [48,49] were administered. At the same time, a semen sample was collected and analyzed to determine semen quality (spermiogram). All parameters were collected three times: at enrolment (t0) and 4 months (t4) and 8 months (t8) after the baseline evaluation. 

The study was carried out in accordance with the guidelines established by the Helsinki Declaration. The Ethic Committees of Southern Campania (29 November 2017, Protocol number 325) and Brescia Province (13 March 2018, Protocol number 2980) and the Italian National Institute of Health (20 December 2017) approved the protocol.

### 2.2. Dietary Assessment

Food intake was assessed at entry into the study (April 2018–January 2019) using a validated food frequency questionnaire (FFQ) to evaluate participants’ diets over the past 12 months [46]. Each subject completed the FFQ with the support of trained nutritionists in an online form.

Daily intakes of macro- and micronutrients plus energy were estimated by linking the frequency and quantities of each food to the Italian Food Tables [50]. The Nova classification [23] was used to categorize each food item/group into four mutually exclusive groups reflecting the extent and purpose of food processing: (1) Group 1 includes unprocessed or minimally processed foods (e.g., fruits and vegetables, meat, milk and fish); (2) Group 2 includes processed culinary ingredients (e.g., honey, butter and oils); (3) Group 3 comprises processed foods with salt, sugar or oil (e.g., canned or bottled vegetables and legumes and canned fish); and (4) Group 4 includes UPFs (e.g., carbonated drinks, processed meat and sweet or salty packaged snacks).

The intake of foods in each Nova group, expressed in grams per day (g/d), and the percentage they represented of the total amount of food eaten were used to estimate a weight ratio that, unlike an energy ratio, takes into account processed foods that do not provide energy (e.g., artificially sweetened beverages), as well as non-nutritional factors related to food processing (e.g., food additives and an altered food matrix). The categorization of individual food items and food groups according to the Nova classification system is available in Supplementary Table S1.

Adherence to a traditional Mediterranean Diet (MD) was appraised by the Mediterranean Diet Score (MDS) developed by Trichopoulou et al. [51]. The MDS was computed by assigning one point to healthy foods (i.e., fruits and nuts, vegetables, legumes, fish, cereals and the monounsaturated-to-saturated fat ratio), whose consumption was above the study population’s median intake; and foods considered harmful (i.e., meat and dairy products) received a positive score if their consumption was below the median. All other intakes accounted for zero points. For ethanol, a consumption of 10–50 g/d corresponded to one point; otherwise, the score was zero. The MDS potentially ranges from 0 (minimal adherence) to 9 (maximal adherence).

### 2.3. Semen Quality Parameters

Semen samples were collected early in the morning through masturbation after 3 days and, at most, 5 days of abstinence. The semen samples were processed immediately and analyzed according to the World Health Organization (WHO) Manual 2010 [52]: a microscope for optical evaluation, with a Makler counting chamber, and two automated semen analyzers (SQA-V GOLD, Medical Electronic Systems Ltd., Petah Tikva, Israel, and the Lenshooke Semen X1 Pro system, Bonraybio Co., Ltd., Taichung, Taiwan), equipped with microscopic integrated optics, were used. Sample volume, sperm concentration, total and progressive motility and the proportion of spermatozoa with normal morphology were assessed.

### 2.4. Statistical Analysis

The main characteristics of the study population across fourths of UPF consumption were presented as mean ± standard deviations (SDs) for continuous variables or numbers and frequencies for categorical traits. Differences in the distribution of baseline covariates were calculated using generalized linear models adjusted for age and energy intake (the GENMOD procedure for categorical variables and the GLM procedure for continuous variables in SAS software, version 9.4).

To investigate the associations between UPFs (the independent variable coded as fourths of consumption) with semen quality parameters (i.e., volume, sperm concentration, total motility, progressive motility and cells with normal morphology modeled as dependent continuous variables), we used beta-coefficients (β) with 95% confidence intervals (95%CI) obtained from multivariable-adjusted linear regression analysis. To account for within-subject correlation due to the repeated measurements of semen parameters taken at three different time points, a hierarchical mixed model approach was employed. This approach allowed for the consideration of both fixed and random effects, thereby providing a robust analysis of the data. Adjusting for within-subject correlation ensures more accurate and reliable estimates of the association of semen quality parameters with UPF consumption.

Potential confounders for inclusion in the analysis models were identified based on the existing literature and theoretical considerations [53].

In addition to the age- and energy-adjusted model, two multivariable models were finally fitted: (i) Model 1 was adjusted for age, energy intake, intervention group, body mass index, waist circumference, physical activity levels and smoking status; and (ii) Model 2 was adjusted per Model 1 but was further controlled for the other Nova groups (the weight ratio modeled as a continuous variable). As a sensitivity analysis, the potential effect of the overall diet quality on the relationship between UPFs and semen parameters was examined by including the MDS in Model 1. Data analysis was generated using SAS/STAT software, version 9.4 (SAS Institute Inc., Cary, NC, USA).

## 3. Results

For the purpose of the present study, of the 147 subjects initially enrolled, 21 participants reporting extreme values of energy intakes (<800 kcal/d or >4000 kcal/d; n = 17) were excluded. Finally, 126 subjects were included in the analyses.

**Figure 1:**
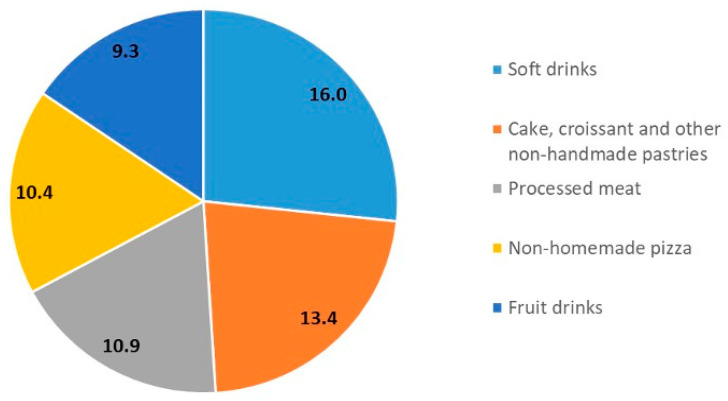
Top contributing foods (%) to the total amount of ultra-processed food consumed in the FASt study.

The mean age of the analyzed sample was 20.0 years (±1.2 years). The average contribution of UPFs to the total food eaten was 20.5% (±9.1). Unprocessed/minimally processed foods, processed culinary ingredients and processed foods contributed 59.2% (±11.1), 2.7% (±0.8) and 17.6% (±6.4), respectively (Table 1). The top five contributing foods to the total UPFs consumed by participants were soft drinks (e.g., carbonated and alcoholic) (16.0%); cake, croissant and other non-handmade pastries (13.4%); processed meat (10.9%); non-homemade pizza (10.4%); and fruit drinks (9.3%) (Figure 1).

The mean values (±SD) of the semen quality parameters were 2.87 mL (±1.37) for volume, 67.2 × 10^6^ cell/mL (±46.8) for sperm concentration, 40.5% (±19.7) for sperm total motility, 27.5% (±17.8) for sperm progressive motility and 6.5% (±4.4) for cells with normal morphology (Table 1).

Compared to the lowest consumption category (Q1), participants with the highest dietary share of UPFs (Q4) reported a lower intake of unprocessed/minimally processed foods and culinary ingredients, as well as a lower MDS, whereas no differences were observed for processed food intake (Table 1). No major differences in demographic and lifestyle factors across levels of UPF consumption were observed.

UPF intake was inversely associated with the consumption of vegetables, fruits and nuts and a lower intake of monounsaturated than saturated fats; participants with high UPFs in their diets also reported lower intakes of proteins, total fats and monounsaturated fats and dietary fibers while having a higher consumption of carbohydrates and sodium than individuals consuming fewer UPFs (Supplementary Table S2).

The associations of UPF consumption with semen quality parameters through hierarchical mixed model analysis are reported in Table 2.

In the fully adjusted Model 2, participants consuming high UPFs (Q4) had a lower sperm concentration (β = −54.16 × 10^6^ cell/ml; 95%CI: −92.91 to −15.40; *p* for trend = 0.0020 across fourths) and progressive motility (β = −14.17%; 95%CI: −28.25 to −0.09; *p* for trend = 0.036) compared to those in the bottom category of intake (Q1). The percentage of normal morphology cells had a tendency to decrease amongst subjects consuming more UPFs compared to the lowest intake (β = −2.93%; 95%CI: −6.22 to 0.37; *p* for trend = 0.071). No associations were observed between sperm volume and total motility (Table 2). The associations between UPFs and semen quality parameters remained substantially unchanged when analyses were controlled for the MDS in replacement of the three Nova groups (Supplementary Table S3); consistent findings were also seen when using data on the sperm parameters at baseline only (Supplementary Table S4).

Values are means ± standard deviations (SDs) unless stated otherwise. The percentage of Nova categories was computed on the total food eaten (the weight ratio). Means and *p*-values were obtained using generalized linear models for both continuous and categorical dependent variables adjusted for age and energy intake.

Data are expressed as regression coefficients β, with 95% confidence intervals (95%CI) obtained from multivariable-adjusted linear regression analyses. Model 1 was adjusted for age, energy intake, intervention group, body mass index, waist circumference, physical activity levels and smoking status. Model 2 was adjusted per Model 1 but was further adjusted for the Nova classification groups, except Group 4 (UPFs).

## 4. Discussion

This cross-sectional analysis carried out in healthy normal-weight young men showed an association between increased UPF consumption and reduced sperm concentration and progressive motility as parameters of the semen quality.

The relationship between UPF intake and sperm quality has been investigated by a few studies, with results consistent with ours. Valle-Hita et al. [43] found a lower sperm concentration and total motility in the highest tertile of UPF intake compared to the lowest in European healthy males aged 18–40 years. They estimated that each 10% increase in energy from UPFs was associated with a 1.50 × 10^6^ decrease in total sperm count. A hospital-based case–control study showed a positive association between UPF intake and the odds of asthenozoospermia in Chinese subjects attending infertility clinics [42]. Two studies considered the consumption of sugar-sweetened beverages (SSBs) as a specific type of UPF. In a Danish cross-sectional study in healthy young men, lower sperm concentrations and total sperm counts were found in subjects with the highest SSB intake compared to non-consumers [41], whereas a US study in healthy young men found that SSB intake was inversely related to total and progressive sperm motility [54].

In addition, studies on dietary patterns could be considered, since specific food groups typical of the Western diet, such as processed meat or sugar-sweetened beverages, are classified as UPFs [55]. In agreement with the UPF studies, dietary pattern studies showed an association between adherence to the Western diet and poor semen quality and increased risk of asthenozoospermia [18,56,57].

Although the exact biological mechanisms are still being explored, several hypotheses provide insights into potential pathways through which UPF consumption might affect semen quality. The first regards UPFs’ nutritional quality. UPFs are often nutrient-poor, meaning they are low in vitamins, minerals, antioxidants and healthy fats, high in added sugars and unhealthy fats and have a high energy density. The deficiency of some nutrients, like zinc, selenium, folate, vitamin C and vitamin E, which are crucial for spermatogenesis, oxidative protection and DNA integrity in sperm, may impair sperm quality [58,59,60,61]. On the other hand, a high intake of trans fats and saturated fats [62,63], as well as a high sugar intake [41,64], has been previously associated with lower semen quality parameters. Moreover, both high fat and sugar intake and low antioxidant intake may induce oxidative stress and the overproduction of reactive oxygen species (ROS) in the body, which can damage sperm DNA, lipids and proteins, thus reducing sperm quality [65]. The second pathway involves the gut microbiome and inflammation grade. UPF consumption could, indeed, negatively impact the gut microbiome, leading to dysbiosis (an imbalance in gut bacteria) [66,67]. Dysbiosis has been linked to increased systemic inflammation, which can negatively affect spermatogenesis and overall semen quality [42,68,69]. Regular consumption of UPFs has been associated with low-grade chronic inflammation [70], which can damage sperm-producing cells in the testes and impair their function [71]. Finally, UPFs could contain some contaminants, such as bisphenols, phthalates and other endocrine-disrupting chemicals (EDCs) found in food packaging and additives [72]. These chemicals can interfere with hormonal regulation of the reproductive system, affecting levels of testosterone and other hormones critical for sperm production and quality, resulting in impaired spermatogenesis [73].

Even though several studies used the percentage of energy intake, in this study, we assessed UPF intake as a weight ratio, i.e., the percentage of UPFs of the total amount of food eaten, in order to also account for processed foods that do not provide energy, in accordance with other authors [30,74]. In our sample, the average contribution of UPFs to the total food eaten was 20.5%, whereas the food category of unprocessed/minimally processed foods had the highest weight contribution (59.2%). The dietary share of UPFs, reported as daily weight or energy contribution, is highly variable in different countries around the world, depending on the most popular dietary pattern: it is generally higher in non-Mediterranean countries, such as the USA, Australia, the UK and Sweden, in which it represents 40–60% of the total energy intake compared to South Europe countries, such as Spain, Romania and Italy, where UPFs contribute for less than 25% of the daily energy intake [74,75]. Indeed, previous evaluations of UPF exposure in the Italian population provided results similar to those from our study. Ruggiero et al. [29] reported an average percentage of energy intake from UPFs of 17.8%, with a higher value in children/adolescents (5–19 years, 25.9%) than in adults (20–97 years, 17.3%). Amongst middle-aged adults from the Moli-sani Study in Italy, UPF consumption (as a weight ratio) was less than 11% of the participants’ diets [31,76]. Among individual UPFs, bakery wares, SSBs, non-homemade pizza and processed meat were the main foods contributing to the total UPFs consumed in these studies [29,31,75], as well as in our study.

Diets rich in UPFs are characterized by low adherence to the Mediterranean diet and, therefore, the low consumption of fruits, vegetables, nuts and legumes. These dietary patterns correspond to a low intake of proteins and fibers and a high intake of carbohydrates, fats and sodium, as reported in several studies [29,31,43]. In accordance with these findings, our results show a lower Mediterranean Diet Score; a lower intake of vegetables, fruits and nuts; lower intakes of proteins and dietary fibers; and a higher intake of carbohydrates and sodium in participants with a high UPF proportion in the diet than in individuals consuming fewer UPFs. However, in our study, no statistical difference was found for energy intake and body mass index (BMI) among the four UPF consumption categories, which may be due to the small sample size.

### Strengths and Limitations

This study has some strengths. First, factors such as lifestyle, overweight/obesity, the presence of chronic diseases and the use of medications or drugs already associated with low semen quality were considered to define rigorous inclusion and exclusion criteria, avoiding confounding effects.

Second, a blinded approach was used to enroll subjects, the semen and fertility status of whom was unknown. Semen analysis was also blinded as regards the subject’s dietary habits. Third, internationally validated tools and methods widely applied in epidemiologic research were used to assess both dietary habits and semen quality.

The main limitation of this study was the relatively small sample size with reduced study power. However, we analyzed more semen samples and questionnaires for many of the subjects, thus increasing the sample size of the study. The use of the EPIC FFQ represents another limitation of this study since it was not specifically conceived to collect dietary data based on food processing, causing possible misclassifications of the UPF exposure, resulting in an underestimation of the effect.

Future research may be improved by including a large number of subjects; measuring other potential confounders, particularly environmental exposures; and adding longitudinal observations over time to take account of changes in participants’ diets and semen quality to better understand the possible cause–effect relationship.

## 5. Conclusions

This study shows an inverse association between dietary UPF intake and sperm concentration and progressive motility in a population of healthy, normal-weight young men. Although the evidence about the impact of diets rich in UPFs on semen quality is still limited, these findings suggest the need to update preventive and interventional approaches aimed at improving semen quality and setting off male infertility, including information about UPF consumption. Further studies are required to confirm these findings, explore the long-term effects on male fertility and reproduction capacity and investigate the biological mechanisms underlying these effects.

## Figures and Tables

**Table 1:** Baseline characteristics of participants from the FASt study (n = 126) across fourths of ultra-processed food consumption.

		Ultra-Processed Food Consumption (Fourths of)				
	All (n = 126)	Q1 (n = 31)	Q2 (n = 32)	Q3 (n = 32)	Q4 (n = 31)	*p*-Value
Unprocessed/minimally processed food (%)	59.2 ± 11.1	69.1 ± 8.8	63.1 ± 5.9	58.8 ± 5.4	45.8 ± 8.2	<0.0001
Processed culinary ingredients (%)	2.7 ± 0.76	2.8 ± 0.81	2.8 ± 0.68	2.8 ± 0.78	2.3 ± 0.64	0.0087
Processed food (%)	17.6 ± 6.4	16.7 ± 8.4	18.0 ± 5.7	16.9 ± 5.1	18.6 ± 6.3	0.66
Ultra-processed food (%)	20.5 ± 9.1	11.3 ± 2.0	16.1 ± 1.3	21.4 ± 1.8	33.3 ± 7.8	<0.0001
Mediterranean Diet Score	4.0 ± 1.8	5.3 ± 1.3	4.1 ± 1.7	3.7 ± 1.8	3.1 ± 1.5	<0.0001
Age (years)	20.0 ± 1.2	19.9 ± 1.1	20.1 ± 1.2	20.1 ± 1.2	19.9 ± 1.3	0.83
Body mass index (kg/m^2^)	22.5 ± 2.4	23.3 ± 2.8	22.3 ± 2.1	22.4 ± 2.4	21.9 ± 2.3	0.16
Waist circumference (cm)	77.2 ± 5.6	77.9 ± 6.1	76.7 ± 6.3	77.5 ± 5.1	76.7 ± 4.8	0.80
Physical activity (IPAQ score)	2222 ± 1655	2645 ± 1701	2053 ± 1599	2318 ± 1809	1872 ± 1463	0.28
Semen parameters						
Volume (mL)	2.87 ± 1.37	3.20 ± 1.36	2.75 ± 1.54	2.81 ± 1.32	2.73 ± 1.26	0.51
Sperm concentration (×10^6^ cell/mL)	67.2 ± 46.8	79.3 ± 57.0	52.2 ± 37.9	71.1 ± 48.1	66.5 ± 40.0	0.13
Total motility (%)	40.5 ± 19.7	43.0 ± 17.9	42.1 ± 22.1	37.1 ± 22.0	40.0 ± 16.3	0.64
Progressive motility (%)	27.5 ± 17.8	31.0 ± 16.0	28.3 ± 19.8	24.4 ± 19.3	26.4 ± 15.9	0.50
Cells with normal morphology (%)	6.5 ± 4.4	7.2 ± 3.8	6.7 ± 4.4	5.9 ± 4.4	6.1 ± 3.8	0.63

**Table 2:** Association of ultra-processed food consumption with semen quality parameters in the FASt study by hierarchical mixed model analysis.

	Ultra-Processed Food Consumption (Fourths of)				
	Q1 (n = 93)	Q2 (n = 96)	Q3 (n = 96)	Q4 (n = 93)	*p*-Value for Trend
Volume (mL)					
Age-adjusted	Ref.	0.27 (−0.51 to 1.04)	−0.16 (−0.93 to 0.61)	−0.36 (−1.14 to 0.42)	0.50
Model 1	Ref.	0.14 (−0.63 to 0.91)	−0.28 (−1.04 to 0.49)	−0.52 (−1.31 to 0.26)	0.30
Model 2	Ref.	0.20 (−0.61 to 1.01)	−0.15 (−1.07 to 0.78)	−0.21 (−1.55 to 1.14)	0.98
Sperm concentration (×10^6^ cell/mL)					
Age-adjusted	Ref.	−29.08 (−51.53 to −6.63)	−8.32 (−30.64 to −14.00)	−7.35 (−29.94 to −15.24)	0.057
Model 1	Ref.	−31.78 (−53.70 to −9.86)	−13.52 (−35.37 to 8.33)	−13.48 (−35.81 to 8.85)	0.025
Model 2	Ref.	−40.06 (−62.75 to −17.36)	−31.66 (−57.78 to −5.55)	−54.16 (−92.91 to −15.40)	0.0020
Total motility (%)					
Age-adjusted	Ref.	1.03 (−9.14 to 11.20)	−0.91 (−10.99 to 9.18)	−1.13 (−11.46 to 9.19)	0.27
Model 1	Ref.	−0.48 (−10.60 to 9.63)	−2.26 (−12.30 to 7.77)	−2.67 (−13.02 to 7.68)	0.18
Model 2	Ref.	−1.86 (−12.36 to 8.64)	−5.04 (−16.62 to 6.54)	−8.77 (−24.84 to 7.29)	0.15
Progressive motility (%)					
Age-adjusted	Ref.	−4.49 (−13.49 to 4.51)	−2.93 (−11.70 to 5.84)	−1.77 (−10.81 to 7.28)	0.25
Model 1	Ref.	−6.09 (−15.01 to 2.86)	−4.37 (−13.07 to 4.33)	−3.70 (−12.74 to 5.34)	0.13
Model 2	Ref.	−8.07 (−17.26 to 1.13)	−8.78 (−18.79 to 1.23)	−14.17 (−28.25 to −0.09)	0.036
Cells with normal morphology (%)					
Age-adjusted	Ref.	−0.90 (−3.00 to 1.20)	−0.07 (−1.99 to 2.14)	0.09 (−2.04 to 2.22)	0.49
Model 1	Ref.	−1.28 (−3.37 to 0.80)	−0.26 (−2.30 to 1.78)	−0.40 (−2.52 to 1.72)	0.30
Model 2	Ref.	−1.77 (−3.91 to 0.37)	−1.33 (−3.67 to 1.00)	−2.93 (−6.22 to 0.37)	0.071

## Data Availability

The data sets generated and/or analyzed during the current study are not publicly available due to the sensitivity of the data but are available from the corresponding author on reasonable request (elisabetta.ceretti1@unibs.it).

## Supplementary Materials

The following supporting information can be downloaded at: https://www.mdpi.com/article/10.3390/nu16234129/s1. Table S1: Categorization of individual food items and food groups according to the Nova classification system. Table S2: Baseline dietary intakes across fourths of ultra-processed food consumption in the population of the FASt Study. Table S3: Sensitivity analysis testing the potential effect of the overall diet quality as reflected by the Mediterranean Diet Score on the relationship between UPFs and semen parameters. Table S4: Association of ultra-processed food consumption with semen quality parameters collected at baseline.
