# Supplementary material for: Consumption of Ultra-Processed Foods and Semen Quality in Healthy Young Men Living in Italy
Source: Nutrients. 2024 Nov 29;16(23):4129. doi: 10.3390/nu16234129 (PMC11644553; doi:10.3390/nu16234129)
Supplement: Supplementary file 1 [file nutrients-16-04129-s001.zip › nutrients-3298745-supplementary.pdf]

## Supplementary Materials

**Article title:** Consumption of Ultra-Processed Foods and Semen Quality in Healthy Young Men Living in Italy
**Author names:** Elisabetta Ceretti, Marialaura Bonaccio, Licia Iacoviello, Augusto Di Castelnuovo, Emilia Ruggiero, Francesco Donato, Stefano Lorenzetti, Danilo Zani and Luigi Montano

**Supplementary Table S1.** Categorization of individual food items and food groups according to the Nova classification system.

| Nova Food Category | Food Items |
|---|---|
| **Group 1: Unprocessed or minimally processed food** | Potatoes; turnip greens and spinach; lettuce; peppers and pumpkin; tomatoes in the main cropping season; tomatoes out of the main cropping season; carrots and beet; cruciferous vegetables; vegetable soups; mushrooms; peas and fava beans; onions; beans, chickpeas, lentils; soybean sprout; citrus fruits; apple, pear, banana, kiwi, grapes, peaches, apricots, plums, strawberries, melon, fruit salad; nuts and dried fruit; whole milk; partially skimmed milk; whole milk yogurt; skimmed milk yogurt; pasta; rice; red meat; white meat; pork; lamb; horse; rabbit; poultry; offal; visible fat portion of meats; beef broth; fish; seafood; eggs; coffee; tea. |
| **Group 2: Processed culinary ingredients** | Vegetable oils; olive oil; extra virgin olive oil; butter; honey; sugar; other fat; other sauces. |
| **Group 3: Processed food** | Soft cheese; hard cheese; mozzarella; white bread, buns and whole-grain bread; canned fish; dried fish; red wine; white wine; rose wine; beer; olives; cured traditional ham; jam; homemade pizza; stuffed pasta; pickled vegetables. |
| **Group 4: Ultra-processed food** | Processed meat; spreadable cheese; sliced cheese; margarine; crispbread and rusks; pizza (not homemade); cakes, pies, pastries and puddings (non-milk based); fruit drinks; ice-cream; stock cube; vegetables pies and deep fried food; fruit yoghurts; dry cakes and biscuits; carbonated/soft/isotonic drinks and diluted syrups; chocolate; breakfast cereals; spirits and brandy; artificial sweeteners; confectionery non chocolate; bread sticks and crackers; mayonnaise and similar; nut spread; soy products. |

**Supplementary Table S2.** Baseline dietary intakes across fourths of ultra-processed food consumption in the population of the FASt Study.

| | **Ultra-processed food consumption (fourths of)** | | | | |
|---|---|---|---|---|---|
| | Q1 | Q2 | Q3 | Q4 | P value |
| N of subjects (%) | 31 (24.6) | 32 (25.4) | 32 (25.4) | 31(24.6) | - |
| Ultra-processed food intake (weight ratio; means ± SD) | 11.3 ± 2.0 | 16.1 ± 1.3 | 21.4 ± 1.8 | 33.3 ± 7.8 | <.0001 |
| **Food groups** (g/d; means ± SD) | | | | | |
| Vegetables | 334.7 ± 160.8 | 232.3 ± 96.1 | 222.8 ± 96.2 | 174.6 ± 79.5 | <.0001 |
| Legumes | 22.8 ± 17.5 | 18.9 ± 16.5 | 17.0 ± 15.7 | 17.2 ± 22.5 | 0.57 |
| Fruits and nuts | 364.1 ± 161.6 | 260.7 ± 150.0 | 209.3 ± 124.4 | 166.3 ± 132.9 | <.0001 |
| Cereals | 220.0 ± 87.2 | 219.3 ± 75.7 | 217.7 ± 96.2 | 210.2 ± 97.3 | 0.94 |
| Fish | 61.4 ± 30.6 | 51.2 ± 25.4 | 47.6 ± 34.4 | 42.9 ± 44.2 | 0.16 |
| Meat and meat products | 214.9 ± 86.9 | 202.9 ± 78.6 | 205.5 ± 71.6 | 210.7 ± 82.7 | 0.92 |
| Milk and dairy products | 215.8 ± 126.0 | 229.7 ± 153.4 | 250.1 ± 184.4 | 177.0 ± 100.0 | 0.17 |
| Monounsaturated to saturated fat ratio | 1.62 ± 0.48 | 1.39 ± 0.21 | 1.31 ± 0.21 | 1.28 ± 0.20 | <.0001 |
| Ethanol | 4.27 ± 5.82 | 4.91 ± 4.27 | 3.79 ± 2.91 | 4.02 ± 3.84 | 0.72 |
| **Macronutrients and nutrients** (%TEI; means ± SD) | | | | | |
| Total carbohydrates | 45.8 ± 6.9 | 47.1 ± 4.3 | 48.3 ± 6.4 | 51.9 ± 6.3 | 0.0010 |
| Protein | 18.2 ± 3.3 | 17.2 ± 2.0 | 16.9 ± 2.6 | 15.9 ± 2.3 | 0.0089 |
| Total fat | 37.6 ± 5.1 | 37.2 ± 3.5 | 36.6 ± 4.6 | 34.2 ± 5.1 | 0.022 |
| Saturated fats | 11.4 ± 2.6 | 12.2 ± 1.8 | 12.4 ± 2.0 | 11.6 ± 2.0 | 0.22 |
| Monounsaturated fats | 17.6 ± 3.0 | 16.7 ± 2.0 | 16.1 ± 2.7 | 14.7 ± 2.6 | 0.0002 |
| Polyunsaturated fats | 9.9 ± 12.5 | 8.5 ± 9.7 | 6.7 ± 10.6 | 8.0 ± 11.5 | 0.74 |
| Fiber (g/d) | 26.5 ± 6.3 | 22.7 ± 6.5 | 21.0 ± 7.6 | 19.8 ± 5.8 | <.0001 |
| Dietary cholesterol (mg/d) | 420.5 ± 97.5 | 430.7 ± 120.2 | 432.0 ± 128.7 | 439.2 ± 142.5 | 0.89 |
| Sodium (mg/d) | 2539 ± 706 | 2749 ± 813 | 2878 ± 939 | 2990 ± 930 | 0.013 |
| Energy intake (kcal/d) | 2634 ± 406 | 2532 ± 610 | 2497 ± 694 | 2682 ± 583 | 0.56 |

%TEI: percentage of total energy intake.

Means and p values were adjusted for age and energy intake.

**Supplementary Table S3.** Sensitivity analysis testing the potential effect of the overall diet quality as reflected by the Mediterranean Diet Score on the relationship between UPF and semen parameters

| | **Ultra-processed food consumption (fourths of)** | | | | |
|---|---|---|---|---|---|
| | Q1 (n=93) | Q2 (n=96) | Q3 (n=96) | Q4 (n=93) | P value for trend |
| *Semen parameters* | | | | | |
| Volume (ml) | | | | | |
| β; 95%CI | Ref. | 0.02 (-0.75 to 0.80) | -0.43 (-1.21 to 0.36) | -0.75 (-1.57 to 0.08) | 0.12 |
| Sperm concentration ($x10^6$ spz/ml) | | | | | |
| β; 95%CI | Ref. | -30.36 (-52.66 to -8.06) | -11.68 (-34.18 to 10.82) | -10.77 (-34.51 to 13.00) | 0.038 |
| Total motility (%) | | | | | |
| β; 95%CI | Ref. | -2.67 (-12.75 to 7.40) | -5.16 (-15.24 to 4.91) | -6.99 (-17.59 to 3.60) | 0.027 |
| Progressive motility (%) | | | | | |
| β; 95%CI | Ref. | -7.85 (-16.80 to 1.12) | -6.59 (-15.37 to 2.20) | -6.86 (-16.14 to 2.43) | 0.026 |
| Cell with normal morphology (%) | | | | | |
| β; 95%CI | Ref. | -1.68 (-3.77 to 0.41) | -0.80 (-2.87 to 1.27) | -1.11 (-3.29 to 1.07) | 0.078 |

Data are expressed as regression coefficients β with 95% confidence intervals (95%CI) obtained from multivariable-adjusted linear regression analyses controlled for age, energy intake, intervention group, body mass index, waist circumference, physical activity levels, smoking status, and the Mediterranean Diet Score.

**Supplementary Table S4.** Association of ultra-processed food consumption with semen quality parameters collected at baseline.

| | Ultra-processed food consumption (fourths of) | | | | |
|---|---|---|---|---|---|
| | Q1 (n=31) | Q2 (n=32) | Q3 (n=32) | Q4 (n=31) | P value for trend |
| *Semen parameters* | | | | | |
| Volume (ml) | | | | | |
| Age-adjusted | Ref. | -0.47 (-1.15 to 0.22) | -0.40 (-1.09 to 0.28) | -0.46 (-1.15 to 0.23) | 0.23 |
| Model 1 | Ref. | -0.52 (-1.22 to 0.19) | -0.42 (-1.12 to 0.29) | -0.48 (-1.20 to 0.24) | 0.26 |
| Model 2 | Ref. | -0.44 (-1.21 to 0.33) | -0.28 (-1.59 to 0.66) | -0.07 (-1.60 to 1.45) | 0.72 |
| Sperm concentration (x$10^6$ spz/ml) | | | | | |
| Age-adjusted | Ref. | -27.01 (-50.27 to -3.75) | -8.16 (-31.41 to 15.08) | -12.76 (-36.16 to 10.62) | 0.62 |
| Model 1 | Ref. | -29.16 (-52.64 to -5.67) | -13.36 (-36.95 to 10.22) | -17.38 (-41.41 to 6.65) | 0.39 |
| Model 2 | Ref. | -37.23 (-62.64 to -11.82) | -30.60 (-61.69 to 0.47) | -57.02 (-107.15 to -6.89) | 0.045 |
| Total motility (%) | | | | | |
| Age-adjusted | Ref. | -2.05 (-11.39 to 7.29) | -6.90 (-16.24 to 2.43) | -3.00 (-12.40 to 6.40) | 0.35 |
| Model 1 | Ref. | -2.73(-12.20 to 6.74) | -6.82 (-16.33 to 2.69) | -3.42 (-13.11 to 6.27) | 0.35 |
| Model 2 | Ref. | -5.30 (-15.69 to 5.09) | -12.04 (-24.75 to 0.67) | -14.74 (-35.24 to 5.76) | 0.067 |
| Progressive motility (%) | | | | | |
| Age-adjusted | Ref. | -3.78 (-12.22 to 4.67) | -7.59 (-16.03 to 0.85) | -4.61 (-13.11 to 3.88) | 0.19 |
| Model 1 | Ref. | -3.92 (-12.57 to 4.73) | -7.56 (-16.25 to 1.13) | -5.06 (-13.91 to 3.79) | 0.18 |
| Model 2 | Ref. | -7.60 (-16.93 to 1.73) | -15.20 (-26.62 to -3.78) | -22.45 (-40.86 to -4.72) | 0.0071 |
| Cell with normal morphology (%) | | | | | |
| Age-adjusted | Ref. | -0.74 (-2.85 to 1.36) | -1.55 (-3.65 to 0.55) | -1.10 (-3.21 to 1.02) | 0.22 |
| Model 1 | Ref. | -0.75 (-2.94 to 1.40) | -1.73 (-3.68 to 0.65) | -1.20 (-3.41 to 1.00) | 0.21 |
| Model 2 | Ref. | -1.61 (-3.94 to 0.71) | -3.36 (-6.21 to -0.51) | -5.29 (-9.88 to -0.70) | 0.014 |

Model 1 was adjusted for age, energy intake, body mass index, waist circumference, physical activity levels, and smoking status.
Model 2 as in model 1 further adjusted for Nova classification groups except group 4 (UPFs).
